# Impacts of local, provincial, and federal immigration policies on health and social services access among women with precarious immigration status

**DOI:** 10.1186/s12889-025-22673-9

**Published:** 2025-04-28

**Authors:** Hanah Damot, Shaina Schafers, Mei-ling Wiedmeyer, Stefanie Machado, Elmira Tayyar, Padmini Thakore, Ruth Lavergne, Shira Miriam Goldenberg, the IRIS team, Stefanie Machado, Elmira Tayyar, Padmini Thakore, Cecilia Sierra Heredia, Yasmin Bozorgi, Eloina Alberto, Zarmina Ali, Sandra Peterson, Ruth Carrillo, Belen Febres-Cordero, Selamawit Hagos, Maggie Hamel-Smith Grassby, Samira Karsiem, Refugio Refugio, Refugio Santos, Germaine Tuyisenge

**Affiliations:** 1 Division of Epidemiology and Biostatistics, School of Public Health, San Diego State University, 5500 Campanile Drive, San Diego, CA 92182 - 4162 USA; 2 Faculty of Health Sciences, McMaster University, 1280 Main Street West, Hamilton, ON L8S 4L8 Canada; 3 Faculty of Health Sciences, Simon Fraser University, 8888 University Drive, Burnaby, BC V5 A 1S6 Canada; 4 Department of Medicine, University of British Columbia, St. Paul’s Hospital, 1081 Burrard Street, Vancouver, BC V6Z 1Y6 Canada; 5 Department of Family Practice, University of British Columbia, 5950 University Boulevard, Vancouver, BC V6 T 1Z3 Canada; 6 Department of Family Medicine, Dalhousie University, 1465 Brenton Street, Suite 402, Halifax, NS B3 J 3 T4 Canada; 7 Division of Infectious Diseases and Global Public Health, School of Medicine, University of California, San Diego, 92093 - 0507 USA

**Keywords:** Immigrant health, Precarious status, Health inequities, Health access, Immigration policy

## Abstract

**Objectives:**

Im/migrant women (e.g., non-status immigrants, refugee claimants, students, temporary foreign workers, visitors, and other migrants) face structural barriers to health and social services access. While immigration is an increasingly recognized social determinant of health, there remains a gap in literature on how structural determinants such as immigration policies and practices (e.g., ‘status-checking’, immigration status) shape im/migrant women’s experiences navigating health and social services. This study aimed to examine the ways in which local, provincial, and federal immigration policies shape health and social services access among im/migrant women with precarious status.

**Methods:**

Between December 2018 and February 2020, we conducted and thematically analyzed qualitative in-depth interviews with im/migrant women (*N* = 51), and service providers (*N* = 10) across Metro Vancouver. Data were collected as part of the IRIS study, which is a community-based, mixed-methods study of im/migrants’ healthcare access prior to and during the COVID-19 pandemic.

**Results:**

Despite policies that purportedly aim to grant access to health and social services in Vancouver regardless of immigration status, participants routinely described ineligibility and fear of detention and/or deportation as pervasive barriers to accessing services, including routine, preventive, and emergency health services, and enrolment of children in schools. Women described social isolation and exclusion as key consequences of federal immigration policies that produced precariousness through temporary and undocumented status. Overall, participants recommended for the elimination of immigration law enforcements and ‘status-checking’ practices in health and social settings.

**Conclusion:**

Sanctuary City policies are recommended to advance im/migrants’ human rights, reduce instances of delayed or denied care, untreated illnesses, and social isolation. Full implementation of Sanctuary principles at the local level (i.e., reduced collaboration between local service providers and federal immigration enforcement) is needed to improve access to health and other services based on need, regardless of immigration status. At the provincial level, elimination of 'status checking' in health settings and expansion of eligibility criteria for health, social, and education programs (e.g., Medical Services Plan, subsidized housing, and BC’s School Act) to include all im/migrants should be considered. At the federal level, increased funding for programs that address inequities in health and social services produced by restrictive immigration policies and ensure pathways to more secure immigration status are recommended. Together, these policy reforms have the potential to address the structural barriers to im/migrant women's health and social services access, and ultimately improve overall public health outcomes.

## Background

Structural determinants refer to the social, economic, and political factors that shape the conditions in which people live, work, and play, collectively known as the social determinants of health [1]. Structural determinants can shape health outcomes by influencing the distribution of resources based on an individual’s social location, such as race, gender, geography, social class, or immigration status [1]. For example, immigration policies are structural determinants that impact im/migrant’s (e.g., non-status immigrants, refugee claimants, students, temporary foreign workers, visitors, and other migrants) access to necessary services, alongside language barriers, system navigation difficulties, and low socioeconomic status [1–13]. While not all access experiences are the same, among im/migrants with ‘precarious status’ (i.e., temporary and non-status residents), fear of deportation is a common barrier to health and social service access that can potentially lead to poor health outcomes [7, 14].

Im/migrants with precarious status lack access to the rights and entitlements associated with permanent residency and citizenship, including Canada’s ‘universal healthcare system’. This creates a misalignment with Canada’s international commitments, including the UN Global Compact for Migration (2018) and UN Declaration of Human Rights (1948) [15–18]. This structural marginalization of im/migrants, arising from the unequal division of opportunities by structures and institutions, has led to their exclusion from several provincial programs essential to health, such as the Medical Services Plan (MSP) and subsidized housing [19–21]. Since im/migrants with precarious status can experience adjustments in immigration status due to factors that are sometimes beyond their control, such as overstaying expired tourist visas and temporary work permits, the refusal of asylum claims, or the cancellation of spousal sponsorships [15], they are at risk of becoming undocumented and excluded from necessary social and health services, thereby denying them access to their fundamental human right to health.

Evidence suggests that the gendered effects of precarious immigration status disproportionately disadvantage women [5, 22]. Canadian studies indicate that im/migrant women with precarious status face greater risks of economic, sexual, and physical exploitation and violence compared to men [5, 22–24]. For example, some women are granted immigration status through a partner’s work permit, while others are tied to a single employer through temporary foreign work programs. This dependence on employers and spouses to maintain their immigration status, and thus their access to social and health services, can make it difficult for some women to leave potentially unsafe situations [5, 22]. There is also evidence that pregnant im/migrant women lack access to prenatal and other necessary services [10, 12, 13, 23–25], which is a critical public health concern posing serious implications for both maternal and child health [26, 27]. Additionally, available literature highlight that im/migrant women face significant barriers to accessing sexual health care, including testing for sexually transmitted infections (STIs) [28, 29]. When immigration policies restrict access to basic health and social services, public health outcomes are adversely impacted, as these policies can hinder public health efforts to promote health and prevent disease across the broader population. While the gendered consequences of precarious immigration status are well documented, there is a scarcity of literature illustrating how im/migrant women’s access to services is affected by immigration policies at various local, provincial, and federal government levels. Understanding these impacts is critical for developing inclusive policies that improve im/migrants’ access to necessary health and social services, which can, in turn, promote overall population health outcomes.

In many US and European jurisdictions, and to a lesser extent, Canada, various local or municipal immigration policies have the potential to influence the implementation and outcomes of federal immigration policy. For instance, Sanctuary City policies, as seen in San Francisco, aim to improve access to public services irrespective of immigration status by providing identification cards to residents [30]. Other US cities that have adopted similar policies include Boston and Seattle [31]. European cities such as Hanover, Milan, and Barcelona have also enforced similar principles, aiming to increase im/migrants’ access to health and social services [32]. In Canada, municipalities have adopted their own ‘status-checking’ practices across various health and social settings; however, full implementation of Sanctuary City principles has yet to occur. Despite the City of Vancouver’s ‘Access Without Fear’ policy, adopted in 2016 with the intent to prevent City staff from contacting the Canada Border Services Agency (CBSA) without an individual’s consent, challenges to implementation persist and im/migrants remain excluded from accessing necessary services [33–36]. Moreover, although the City of New Westminster introduced a similar policy in 2022 [37], other cities in the Metro Vancouver region have yet to follow, further limiting im/migrant women’s access to services.

Currently, there is limited information on how structural determinants of immigration policies and practices affect im/migrant women’s experiences accessing (or attempting to access) health and social services, particularly from the perspectives of im/migrant women with precarious status, whose lived experiences remain poorly documented in Canada. As such, our objective was to describe how im/migrant women with precarious status’ access to health and social services is shaped by local, provincial, and federal immigration policies and practices in Metro Vancouver, Canada.

## Methods

### Study design

Evaluating Inequities in Refugee and Immigrants’ Health Access (IRIS) is a community-based mixed-methods study partnered with local im/migrant-serving organizations (e.g., MOSAIC, PIRS, Sanctuary Health, Watari), a multilingual im/migrant advisory board (English, Dari, Farsi, Spanish, Tigrinya), and guided by a diverse, interdisciplinary research team with lived experiences of migration and expertise in qualitative and health services research. As previously described [38], IRIS is rooted in principles of community-engaged research. Im/migrant community members and participants were actively involved across all stages of the research, collaborating with the IRIS team through initial consultations and focus groups, on the development of research priorities, research questions, data collection, recruitment strategies, data analysis, and interpretation and dissemination of data. This participatory approach helped mitigate the IRIS team’s biases and assumptions by grounding the research in the unique lived experiences and needs of the community, rather than their own experiences of migration. The IRIS team was comprised of women principal investigators, research assistants, community associates, coordinators, and graduate students. The reporting of this study conforms to the Consolidated criteria for Reporting Qualitative Research (COREQ) guidelines.

### Study setting

This study is set in Metro Vancouver, BC, Canada. Canada hosts a diverse and multicultural im/migrant population, with immigrants comprising 23% of its overall population in 2021 [39, 40]. While estimates of undocumented im/migrants are scarce, existing data suggests a range between 200,000 to 500,000 [41], with many residing in urban centres [5, 15, 42, 43]. Of Canada’s overall immigrant population, 17% reside in BC, equating to a total of 1,425,710 people [40]. In recent years, BC’s immigrant population has almost doubled, with 38,085 having arrived in 2016 and 69,470 in 2021 [40]. In Metro Vancouver specifically, 41.8% of the overall population were immigrants and 5.1% were non-permanent residents in 2021, with the top four primary countries of origin being China, India, Philippines, and Hong Kong [44]. Primary languages spoken include Mandarin, Cantonese, Punjabi, and Tagalog [44]. The highly multicultural and overrepresentation of im/migrants locally highlight the need for the development and implementation of inclusive immigration policies.

In Canada, the enforcement of federal immigration law (i.e., detainment and deportation of im/migrants), primarily falls onto the CBSA in collaboration with the Royal Canadian Mounted Police and other local police agencies [39, 45, 46]. In 2016, Vancouver passed an ‘Access Without Fear’ policy with the intent to provide access to municipal services independent of im/migration status. The City committed to working with other jurisdictions, including Health Authorities, to protect the confidentiality of patient information and create zones free of immigration enforcement in settings where people seek assistance [33].

### Data collection

From December 2018 to February 2020, we conducted in-depth one-on-one interviews at our community office with im/migrant women with precarious status (*N* = 51) and people who provide health and social services to im/migrants (*N* = 10) across Metro Vancouver, as previously described [47]. Snowball sampling was used to select participants. The IRIS study was advertised through community partners and organizations, where research team members conducted information sessions and poster presentations. Community partners and participants of these sessions were asked to share information about the IRIS study with their networks.

Im/migrant women aged 15–49 years who self-identified as a woman (cis or trans), moved to Canada from another country, and able to provide informed consent were eligible for the study. Service providers were eligible if they were employed in the health, social or legal sectors with im/migrant women and able to provide informed consent.

Trained interviewers with lived migration experience conducted interviews and focus groups in Farsi, Dari, Spanish, and English. Open-ended questions related to migration history, settlement into Canada, experiences with health and social services in Canada, and recommendations for improving health service access were asked during interviews. All sessions were conducted in each participant’s preferred language, audiotaped, and ranged from approximately 45 min to 2 h depending on the participant’s comfortability or until data saturation was reached. Field notes were completed during and after the interviews. All im/migrant women received an honorarium of C$40 and were offered referrals to community-based social, health, and legal supports as needed.

Our study primarily included racialized women with precarious im/migration status, and therefore does not capture the experiences of women in more secure immigration categories. Interviews with service providers elicited their professional experiences working with im/migrant women, gaps in services, and perspectives on how to improve im/migrant women’s health and wellbeing. A socio-demographic questionnaire was administered to interviewees.

### Data analysis

This analysis is restricted to data collected before March 2020 to explore im/migrant women’s access to health and social services prior to the service delivery changes that occurred due to the Covid-19 pandemic. Interviews were transcribed verbatim and simultaneously translated into English, and then checked for accuracy. Unique codes were used to identify participants, with personal identifiers removed. Coding and management of data was conducted using NVivo v12 (QSR, AUS). Content analysis was used to generate initial codes related to im/migrant women’s experiences with health and social services. A team of four coders iteratively adapted the coding scheme, concurrently with the analysis of initial transcripts, and collaborated to establish a shared understanding of the codes. Further steps of the analysis were completed by the first two authors, guided by Braun and Clarke’s reflexive thematic analysis [48]. The analysis involved reviewing codes pertaining to women’s health and social services access, focusing on emerging themes related to the lived experiences of women with precarious status in Metro Vancouver and how access to health and other services is shaped by structural determinants such as immigration policies and practices (e.g., Sanctuary City policies and ‘status-checking’ practices in local settings), eligibility requirements of provincial programs (e.g., MSP), and federal immigration laws enforced by the CBSA. The WHO’s Commission on Social Determinants of Health (CSDH) conceptual framework was used as a theoretical lens to analyze im/migrant women’s barriers to accessing health and social services [1]. This framework focuses on the key role of social determinants of health in achieving health equity by highlighting how structural determinants, such as immigration status, influence health outcomes [1]. As the emergent data and our framework highlight that immigration policies and their implementation at the local, provincial, and federal levels can be critical social determinants of im/migrant women’s access to health and social services, in the final step of the analysis we identified four key crosscutting themes which form the basis for our results: *1) Fear of accessing services due to immigration-related policies and implementation practices; 2) Gaps and delays in health services access due to fear and ineligibility for provincial health insurance; 3) Social isolation and exclusion due to fear and ineligibility for provincial health and social programs; and 4) Recommendations for policies and practices to increase access*. Minor themes related to social determinants of health were also identified through an iterative coding process, offering deeper insights into participants' experiences of fear, exclusion, and social isolation when accessing health and social services. Preliminary results were shared for ‘member checking’ with participants and regular meetings were conducted with community partners to discuss and confirm our interpretation of data.

### Ethics statement

IRIS holds ethical approval from the Simon Fraser University and the Providence Health Care/University of British Columbia harmonized ethics review boards. All procedures were conducted in accordance with the ethical standards expressed in the Declaration of Helsinki. Written informed consent prior to participation was obtained from all participants, and extensive confidentiality protocols were followed.

## Results

### Participant characteristics

Among the 51 im/migrant women included in this analysis, women’s median age was 30.5 (range: 18–42) and the average duration living in Canada was 2.5 years (range: 0.4–12 years) (Table 1). Latin America was the primary region of origin. Most participants belonged to multiple im/migration categories since arriving to Canada, including temporary residents and refugee claimants. Providers were a diverse group in terms of roles (e.g., community outreach workers, nurse practitioners, physicians) and country of origin (Table 2). All providers identified as women.

**Table 1:** Im/migrant Women: Participant Characteristics (*N* = 51), 2018–2020

**Variable**	
Age, *years*^a^	30.5 (18–42)
Region of origin	
• Latin America	47
• Europe	2
• Middle East	2
Primary language	
• Spanish and English	2
• Spanish only	47
• Farsi	2
Time in Canada, *year*^a^	1.5 (0.4–12)

Data are N (#) of women, unless otherwise indicated

^a^Median (range

**Table 2:** Service Providers: Participant Characteristics (*N* = 10), 2018–2020

**Variable**	
Age, *years*^a^	43 (33–57)
Gender	
• Woman	10
• Man	0
• Non-binary/Other	0
Region of origin	
• Canada	3
• Asia	2
• Latin America	2
• Middle East	1
• Europe	1
• Prefer not to say	1
Years working with im/migrant women, *years*^a^	7 (2–30)
Roles	
• Community advocate/outreach worker	2
• Nurse/Nurse Practitioners	4
• Settlement worker	2
• Other (e.g., social worker)	2

Data are N (#) of women, unless otherwise indicated

^a^Median (range)

Overall, participants’ interview narratives revealed three main themes of how current local, provincial, and federal immigration policies and practices impact their experiences accessing health or social services. Many participants experienced fear of accessing services due to immigration-related policies and implementation practices (theme 1). This fear, along with ineligibility for many provincial health and social programs, led to gaps and delays in accessing health services (theme 2), and social isolation and exclusion (theme 3). Participants also provided recommendations for policies and practices to improve access to health and social services (theme 4).

### Theme 1: Fear of accessing services due to immigration-related policies and implementation practices

Current federal immigration policies relegate migrants to precarious immigration categories, leaving them without access to numerous health and social services. To reduce these consequences, local level responses that adapt Sanctuary City principles are urgently needed. However, as most health and social services are operated by provincial entities, municipal policy implementation across various establishments is limited. As a result, participants’ access experiences may differ based on jurisdiction. For instance, while the cities of Vancouver and New Westminster have approved similar Sanctuary City policies, committed implementation is lacking and yet to be adopted across the Metro Vancouver region. One participant, who initially arrived with a tourist visa, recalled how losing immigration status caused severe repercussions with regard to their access to services:

*We were scared, nervous about all of the services that we could be denied from. We lost our status after one week. After that, our lives are limited… the fear is there when you lose your status (Woman, age 23)*

Many participants were questioned about immigration status as part of intake procedures in health establishments. Women had heard of situations where immigration authorities were alerted of a person’s undocumented immigration status in health settings and often feared that their information would be shared with immigration officials (CBSA) and/or police, potentially leading to detainment and/or deportation. While it is likely that intake clerks inquire about immigration status to assess health care entitlements (e.g., public health insurance), not necessarily to alert immigration authorities, participants explained how ‘status-checking’ contributes to a climate of fear that discourages them from seeking medical care.

Fear of deportation is a barrier to service access that is unique to im/migrants with precarious status and is further exacerbated by factors such as language preference. Although ‘status-checking’ practices can add to this fear, participants were persistently reminded of the potential ramifications of being reported to federal immigration authorities in their interactions with others. One woman who arrived in Canada five months pregnant and lost her tourist visa after one week, did not seek nor receive care until her eighth month of pregnancy. She explained how despite the absence of ‘status-checking’ practices, her fear of deportation worsened at the thought of having to use an interpreter, as it drew “*more attention*” to her immigration status rather than “*receive medical attention*”.

For many women, fear of deportation is intensified by the prospect of returning to their home countries, where they would face violence, extortion, impoverishment, and other conditions they desperately wanted to escape. One woman who experienced abuse and costly immigration processes, lost status when her workplace unexpectedly opted to send workers home. She contemplated the decision to stay in Canada despite the challenges associated with being undocumented:

*Having all that debt in Guatemala, how could I go back? How would I pay? From where? I can’t… So you make some decisions to stay (Woman, age 31)*

Participants also identified police presence as a barrier to health services access. Although the responsibility for enforcing immigration law in Canada falls onto federal agencies (e.g., CBSA and Immigration, Refugees and Citizenship Canada), participants were suspicious that police collaborate to report undocumented im/migrants. In one example, a woman without status quickly left the hospital without treatment due to police being “*all over the place*” and the intake clerk asking “*for papers*” in a loud voice.

### Theme 2: Gaps and delays in health services access due to fear and ineligibility for provincial health insurance

Fear of immigration enforcement in local health settings and exclusion from provincial health entitlements (e.g., MSP) often resulted in the delay or avoidance of necessary health care for routine, preventive, and emergency services, which potentially led to negative health outcomes. For example, a woman who went through a period without status but is now a permanent resident, recounted how ‘status-checking’ practices and health insurance ineligibility ultimately led to the avoidance of necessary medical care and increased risk for pregnancy complications:

*I had a kidney infection, and I was pregnant… I spent the whole night in pain… But I couldn’t go, because the first thing they ask… “are you a visitor? a resident? What is your status? Where is your medical card?” (Woman, age 32)*

In conjunction with barriers to accessing health services related to precarious status, such as ineligibility for public health insurance and financial constraints, women experienced alarming gaps in access to sexual and reproductive health (SRH) and other health services even in emergency situations that could be life-threatening. A woman who is now a resident, but whose Canadian work visa expired while pregnant explained:

*You prefer to let yourself die at home… because if you go to a hospital, you feel that you are going to lose everything… (Woman, age 37)*

Many women had first prenatal visits at 6 or 7 months into pregnancy and lacked access to services such as contraception and testing for STIs, due to fear of being asked about their immigration status at point of service. This fear likely stems from the absence of Sanctuary City principles in health settings, which eliminate the need for ‘status-checking’ practices. One woman, who was working on claiming refugee status, explained the implications of her precarious status with regard to pregnancy care:

*It is not easy at all to find a place where they can see you, or that they help you to monitor your pregnancy… It is very expensive, and… sometimes they focus too much on the person’s status. (Woman, age 19)*

Another woman, who initially came without status but was in the process of applying for permanent residency, recalled how she resorted to over-the-counter medication to relieve her chest pain rather than seek professional care due to concerns regarding immigration enforcement in local hospitals:

*They had told us that if we went to a hospital, they would immediately inform immigration, or the police that I didn’t have documentation… (Woman, age 32)*

In contrast, immigrant-friendly health providers and clinics that took measures such as offering free services or special programs for women who lack provincial health insurance or accept out-of-pocket payments without inquiring about immigration status were described as safer, more accessible, and more positive healthcare experiences. One woman described the impact of finally being treated for her health issues, after being referred to a community health centre she could access without fear:

*I told them that I was so weak I didn’t want to get out of bed, that I didn’t want to do anything, that I felt that my life was nothing, that I couldn’t take it anymore, that everything hurt, and they sent me some tests, and they realized that my iron was extremely low. They realized that. (Woman, age 37)*

### Theme 3: Social isolation and exclusion due to fear and ineligibility for provincial health and social programs

Although many women lose status for reasons beyond their control (e.g., immigration scams, leaving abusive work/intimate relationships, employment lay-offs, administrative errors, or delays), the fear associated with being undocumented led to social isolation and negative impacts on psychological well-being due to the absence of trustworthy social networks and supports. When asked what advice she would give to another im/migrant woman in her situation, one woman said:

*Ask, speak, don’t remain silent, and don’t be afraid… sometimes because of fear, you don’t look for help, you don’t talk to people, or sometimes because you are ashamed or because you are going to be judged or critiqued for what we are going through. (Woman, age 31)*

An outreach worker explained that many undocumented women fear leaving the home altogether to avoid being asked questions about their status and potentially detained or deported. Due to the fear of being identified when accessing community services and lack of more formally available supports, many participants face social isolation and thus are further excluded from the community due to limited social networks and access to resources. To foster social connection and obtain information about accessible services, some participants have resorted to social media. In these virtual settings, im/migrants were able to access and provide support to one another, share their concerns about their immigration status, and ask questions about available services, without the fear of being deported or detained.

Participants also spoke about how precarious immigration status led to exclusion from social supports, significantly impacting their health and social development. In BC, access to most provincial supports are determined by immigration status (e.g., MSP and Affordable Child Care Benefit), with the exception of educational programs under the BC School Act, as eligibility is determined by boards of education [49–51]. Without access to school and child care due to marginalization and high costs, families with precarious immigration status are left socially isolated. One im/migrant mother described how undocumented status led to both the social exclusion and isolation of her and her son:

*I cannot have access to health, or the school, for my son… right now he is at a stage where I should at least have him at least in a preschool… so that he is not alone at home. (Woman, 26)*

Im/migrant women with precarious status are also excluded from programming offered by settlement organizations and many community-based organizations (CBOs). Additionally, they face increased vulnerability, such as being forced to work low paying jobs, exploitation in the workplace (e.g., not being paid for their work), and difficulty finding housing (e.g., ineligibility for subsidized housing under BC Housing) [21]. Overall, access to many aspects of life in Canada is reserved for citizens and permanent residents, as revealed in the following quote:

*You don’t know what things you can do or how much access you can have as a person… being here, regardless of your status. (Woman, age 31)*

As would be expected, fear to access services was alleviated for women who gained status. One woman described a symbolic shift associated with status change that allowed her to participate in society more fully:

*Once I became a permanent resident, I think it’s also psychological. You feel that you finally belong (Woman, 27)*

### Recommendations for policies and practices to increase access

To mitigate access barriers related to fear, women and providers often shared information with one another about where patients have experienced ‘status-checking’ and which establishments to avoid. This suggests the need to eliminate inquiries on immigration status in health and social settings. Participants also identified CBOs as crucial supports in creating dialogue amongst im/migrant women, as well as providing them with information and referrals to services they can access without fear, such as trusted midwives, health clinics who have taken steps to create welcoming and safe environments, and other services such as food hampers, donations, and legal support.

Many participants remained positive and hopeful for change when asked about suggestions on how to increase im/migrant women’s health and social services access. Participants emphasized program and policy reform to address the social inequities perpetuated by current immigration policies. Collaboration between fellow im/migrants and community members in shaping existing policies to ensure that “*status is not a limit*” that impedes service access was recommended. In particular, greater advocacy among community members was encouraged to promote policies that allow im/migrants to access services without fear and “*that they fight more so that the government does not discriminate*”. They also endorsed information sharing to increase service access:

*Residents or citizens should be able to give [im/migrants information] about where to go… It should not only be paisanos [other Mexicans] or others that already have the need or have had them, but also others that are from here should be aware. (Woman, age 23)*

Other recommendations include the implementation of a temporary and confidential medical card for im/migrants that could be helpful in facilitating health care access, and in turn, reduce instances of delayed or denied care, untreated illnesses, and social isolation among undocumented im/migrants or those awaiting permanent residency:

*With that card… maybe you don’t have to give your name… and… with a small fee, that you pay a certain amount each month, until you can… adapt… your status (Woman, age 32)*

Overall, to facilitate access to necessary services and reduce the harms associated with the fear of deportation, women and providers stressed the need for health and social settings that are free of immigration law enforcement. Eliminating ‘status-checking’ practices is essential in reducing im/migrant women’s fear of deportation, improving their health, and upholding their human rights.

## Discussion

In this study, we examined the experiences of im/migrant women in accessing health and social services in Metro Vancouver, particularly with respect to the interplay of local or municipal, provincial, and federal immigration policies and practices. Key findings highlighted how im/migrants’ fears of detention and/or deportation significantly impeded their health care utilization. This fear, along with ineligibility for many provincial health and social supports, led to gaps and delays in accessing health services, as well as social isolation and exclusion. The results of this research underscore the compelling need for local immigration policy reform to ensure that im/migrant women and their families can access services safely, and without fear of negative immigration or law enforcement consequences. Provincially, changes such as the elimination of ‘status-checking’ practices within health and social services is recommended. At the federal level, changes to immigration policies and enforcement practices are needed to reduce precarity faced by im/migrants to Canada, including by providing more widespread pathways to permanent residency.

Inquiries on immigration status and sometimes reporting to immigration officials, by workers in health settings produces a climate of fear among im/migrant patients. Consistent with other qualitative studies from the US and Canada, highlighting fear of detention and/or deportation as a major barrier to health care access among im/migrants [7, 52–54], our findings suggest that the fear and avoidance of accessing health services among im/migrant women is largely due to ‘status-checking’ and police presence in health settings. Participants reported that the presence of immigration officials and/or police in health settings contributed to their fear of being identified and significantly influenced their decision to leave hospitals and cancel medical treatments, despite their need for care. Previous qualitative studies exploring the barriers to health access among im/migrants noted similar findings; however, this study is the first to explore im/migrant women’s lived experiences within the Metro Vancouver context [7, 55].

In this study, fear of detention and/or deportation led to severe gaps and delays in access to essential health and social services among im/migrant women. Many participants avoided critical sexual and reproductive health and other emergency services due to fear of being deported back to their home countries, where they may face dangerous and unstable environments. This is consistent with findings from other quantitative and qualitative studies from the US, Europe, and Canada, which detail how fear of detention and/or deportation has greatly contributed to im/migrant’s avoidance of necessary care, limiting public health efforts to improve overall population health outcomes [6, 27, 56].

The impacts of fear on health, and its relationship to social isolation and exclusion, were also identified. The climate of fear produced by ‘status-checking’ practices has led to social isolation and exclusion among im/migrants, resulting in negative psychological well-being. Along with avoidance of healthcare, some participants expressed their fear of accessing municipal services while others avoided leaving their homes altogether, further limiting their access to information on available community supports. The social exclusion of im/migrant families has also led to a lower standard of living, thus contributing to poorer health, as they are excluded from well-paying jobs, proper housing, enrolment in schools, and community programs. This is supported by previous work from Canada and the U.S. indicating the critical implications of immigration status in shaping access to services and concomitant health and social sequelae [5, 7, 11, 23, 24, 26, 57–61]. Previous research also shows that im/migrants with precarious status face increased social isolation and negative health outcomes due to anti-immigration policies and the increased involvement of immigration enforcement in health settings [56, 61], further emphasizing the impact of immigration policies as structural determinants of health.

Despite the City of Vancouver’s ‘Access Without Fear’ policy, audits by community groups discovered inconsistencies in implementation, as many City staff were unaware of the policy change [35, 62]. Although recommendations were proposed, including improved staff awareness, engagement, training and support, and creation of guidelines to bridge the ongoing implementation gaps, [62–64] policy implementation remained incomplete as of 2022 [35]. These significant gaps to implementation have eroded trust among im/migrants, preventing them from accessing critical living, safety, emergency housing, and community services [35]. The lack of implementation of Sanctuary City principles within local policies is likely due to communication and training gaps arising from jurisdictional limitations. As many health and social services are operated at the provincial level, municipalities are unable to enforce policies in local settings, resulting in the exclusion of im/migrants from essential health and social services [21, 65].

### Recommendations

Findings suggest the urgent need for rights-based policy and practice reforms at local and provincial levels. To promote an equitable distribution of resources among im/migrants, committed implementation of Sanctuary City principles across all cities is critical in deterring collaboration between local establishments and federal immigration authorities. Municipalities can refer to Sanctuary City examples from Europe and the US to address structural barriers to health and social services access faced by im/migrants at the policy level [32]. The provincial government can support Sanctuary City policies in social and health settings by eliminating ‘status-checking’ practices and expanding eligibility criteria for provincial programs and policies, such as the Medical Services Plan and BC School Act, to specifically include all im/migrants. In the absence of these policies, improved training for frontline workers to provide services without inquiring into people’s immigration status is imperative. Our findings build on existing evidence suggesting that community engagement and supports, as well as policies that discourage ‘status-checking’ practices can help facilitate im/migrant’s access to health and social services [7, 29, 55].

At the federal level, increased funding is required to scale-up im/migrant-serving organizations and municipal services that facilitate pathways to a more secure immigration status and improve im/migrants’ social determinants of health. Municipalities in BC can follow Sanctuary City examples from European cities that provide im/migrants with access to legal counseling, health care access, housing and welfare support [32]. These services are urgently needed, especially for im/migrant women facing financial constraints and housing insecurity due to abuse and power exerted by partners, employers, landlords, and other individuals. Such circumstances significantly contribute to increased stress, impeding many im/migrant women’s ability to improve their health or precarious situations [61, 66–68]. Expanding support for im/migrant women can also prevent negative intergenerational effects. For im/migrant mothers with caretaking responsibilities, for example, securing and maintaining employment can be particularly challenging, as jobs often require additional overtime hours [68–73]. Scaling up these services can help address the social inequities created by structural determinants such as immigration policies, and that make it difficult for community-based organizations to connect im/migrant women with necessary health and social supports.

### Strengths and limitations

Research on im/migrant women with precarious status remains limited. A strength of this community-based study is its ability to capture the unique experiences of an underrepresented population regarding health and social service access within the Metro Vancouver context. However, as this study primarily focuses on the lived experiences of im/migrant women with precarious status, the gendered experiences of men and non-binary individuals are not captured. Additionally, since recruitment was conducted through snowball sampling, perspectives of im/migrants who lack social networks or access to community-based organizations were also not represented.

## Conclusion

Findings of this study illustrate how policies and practices at multiple levels of government interact to affect im/migrant women’s health and social service access. Im/migrant women in Metro Vancouver face unique barriers accessing health care and social services, including the fear of deportation and ineligibility for various provincial health and social programs that lead to social isolation/exclusion as well as gaps and delays in necessary services. This research calls upon the urgent need for full implementation of local Sanctuary City policies and inclusion of im/migrants in provincial supports to improve im/migrants’ access to health and social services. Increased federal funding to scale-up programs that facilitate pathways to a more secure immigration status and mitigate the social class inequities produced by existing immigration policies are also required.

## Data Availability

The datasets used and/or analysed during the current study are available from the corresponding author on reasonable request.
